# Risk and protective factors associated with brain grey matter patterns in a population-based cohort of cognitively unimpaired 70-year-olds

**DOI:** 10.21203/rs.3.rs-7349116/v1

**Published:** 2025-08-27

**Authors:** Giulia Lorenzon, Anna Marseglia, Konstantinos Poulakis, Camillo Imbimbo, Lina Rydén, Evangelos Galaris, Olof Lindberg, Sara Shams, Rosaleena Mohanty, Daniel Ferreira, Miia Kivipelto, Maria Eriksdotter, Silke Kern, Ingmar Skoog, Eric Westman

**Affiliations:** Karolinska Institutet; Karolinska Institutet; Karolinska Institutet; Karolinska Institutet; University of Gothenburg; Karolinska Institutet; Karolinska Institutet; Karolinska University Hospital, Karolinska Institutet; Karolinska Institutet; Karolinska Institutet; Karolinska Institutet; Karolinska Institutet; University of Gothenburg; University of Gothenburg; Karolinska Institutet

**Keywords:** Brain ageing, Biomarkers, Cardiovascular risk factors, Dementia, Alzheimer disease, Prevention

## Abstract

**Background:**

Aging involves heterogeneous brain grey matter (GM) loss patterns that may overlap with dementia-related changes. We evaluated cognitively unimpaired older adults to identify specific GM patterns, their clinical and cognitive profiles, and longitudinal trajectories.

**Methods:**

We analyzed 746 participants from the Gothenburg H70 Study using random forest clustering based on MRI measures of cortical thickness and subcortical volume across 41 regions. We examined associations with clinical and MRI variables, APOE status, and CSF Alzheimer biomarkers (n = 286), and assessed 5-year cognitive and brain trajectories using regression models.

**Results:**

Five clusters emerged, mainly differing in frontoparietal regions. Compared to Cluster 1 (reference), Cluster 2 showed diffuse GM loss, higher odds of diabetes (OR = 2.54, 95%CI [1.27–5.06]) and risk alcohol consumption (OR = 1.83, 95%CI [1.13–2.97]), poorer episodic memory (β=–0.190, p = 0.014) and visuospatial abilities (β =–0.209, p = 0.044), and greater longitudinal decline in MMSE (β_slope_ = − 0.448, p = 0.035) and increase in white matter hyperintensity volume (β_slope_ = 1.843, p = 0.004). Clusters 3 showed thicker GM and lower BMI (OR = 0.57, 95%CI [0.35–0.94]). Cluster 4 had preserved GM, lower smoking habits (OR = 0.62, 95%CI [0.40–0.95]), triglycerides levels (OR = 0.55, 95%CI [0.32–0.95]) and depression (OR = 0.17, 95%CI [0.05–0.56]), higher education (OR = 2.52, 95%CI [1.08–5.87]) and better cognition in multiple domains. Cluster 5 had mixed GM pattern and higher odds of heart disease (OR = 3.44, 95%CI [1.48–8.01]).

**Conclusions:**

Cardiovascular and psychosocial factors are key determinants of GM integrity and cognition. Targeting these risks factors may preserve brain health in late life.

## Background

Dementia represents a major public health priority, affecting over 55 million people worldwide [1]. With projections expected to triple in the next 30 years, prevention strategies are essential, as emerging evidence suggests that up to 45% of cases could be avoided by targeting modifiable factors [2]. However, early detection of at-risk individuals remains challenging. Identifying clinical risk profiles associated with pathological brain and cognitive aging in still asymptomatic individuals is key to enable timely and targeted prevention strategies.

Structural magnetic resonance imaging (MRI) studies have shown that aging is associated with heterogeneous patterns of brain grey matter (GM) loss, particularly in frontal and parietal lobes, as well as with reductions in white matter (WM) volumes [3]. Considerable interindividual variability exists, and genetic, cardiovascular and lifestyle-related risk factors may accelerate these processes [4–6]. These GM and WM changes are thought to underlie the cognitive decline typically seen in aging, especially in processing speed and executive functioning [3]. In contrast, neurodegenerative diseases associated with dementia exhibit more pronounced and specific patterns of cortical atrophy [7]. In Alzheimer’s disease (AD), distinct and consistently observed subtypes include typical, limbic-predominant, hippocampal-sparing, and minimal atrophy patterns [8, 9]. Other characteristic atrophy subtypes have been identified in Parkinson’s disease (PD) [10] and dementia with Lewy bodies (DLB) [11]. Importantly, these changes often precede cognitive decline [12], and characterizing their biological mechanisms in cognitively unimpaired individuals may clarify the transition from non-pathological to pathological brain aging.

MRI studies employing data-driven clustering approaches have increasingly been used to explore brain morphological heterogeneity in cognitively healthy older adults, revealing subgroups with distinct biological, cognitive, and clinical profiles [13–15]. In our previous work, we applied longitudinal Bayesian clustering to identify frontoparietal GM atrophy patterns and trajectories that were differentially associated with cerebrovascular burden and cognitive decline [16]. However, relatively few longitudinal studies have examined these associations in non-clinical and demographically uniform populations [12, 14].

In this study, we examined a cohort of cognitively unimpaired older individuals from the general population with the aim to: (1) identify specific cortical and subcortical grey matter patterns; (2) investigate their associations with clinical and brain MRI variables, *APOE* status and CSF AD biomarkers; and (3) assess how these subgroups evolve over a 5-year follow-up.

## Methods

### 2.1. Study design

**Figure 1:**
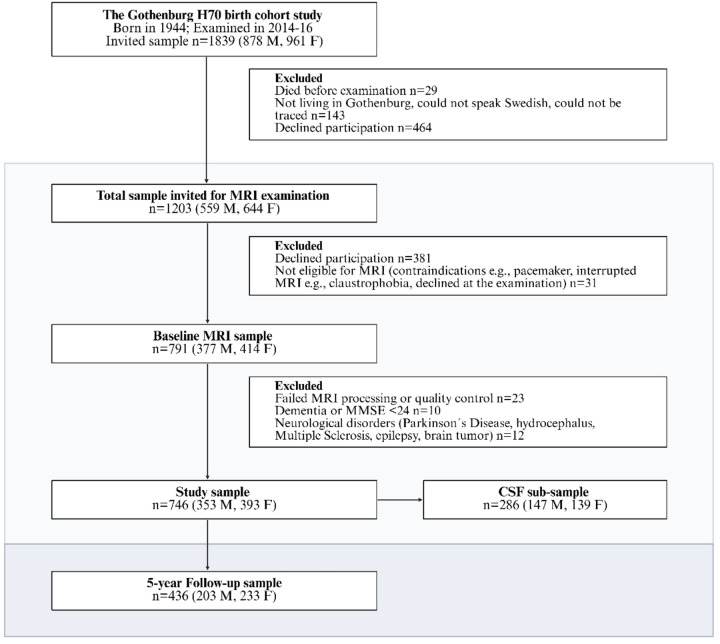
Flowchart of the study sample. The diagram illustrates the selection process from the original Gothenburg H70 birth cohort to the final study sample, including exclusions, the CSF subsample, and the 5-year follow-up group. Abbreviations: M, males; F, females; MRI, magnetic resonance imaging; CSF, cerebrospinal fluid.

This study utilized data from participants in the Gothenburg H70 Birth Cohort Study (H70–1944), a Swedish population-based study comprising individuals born in 1944 [17]. Between January 2014 and December 2016, 791 out of 1,203 eligible 70-year-olds (65.8% response rate) underwent brain MRI (see Fig. 1). After excluding participants due to imaging quality issues (n = 23) and neurological disorders (n = 22), the final sample included 746 cognitively healthy individuals (353 men, 393 women). Cerebrospinal fluid (CSF) samples were available for 286 of these participants.

At the 5-year follow-up (age 75), 436 individuals (58,4% of the whole sample) returned for re-evaluation. The H70 study received ethical approval from the Regional Ethical Review Board in Gothenburg. Written informed consent was obtained in agreement with the Helsinki Declaration.

### 2.2. Brain MRI acquisition and preprocessing

Participants were scanned on a 3.0T MRI scanner (Philips Medical Systems). The imaging protocol included the following sequences: three-dimensional (3D) T1-weighted Turbo Field Echo (TFE), T2-weighted, fluid-attenuated inversion recovery (FLAIR), diffusion tensor imaging (DTI), and venous BOLD (VenoBOLD). For detailed MRI acquisition parameters see Rydberg et al. [17]. All images were processed and stored through the-Hive database system at Karolinska Institute [18], and underwent quality control as described previously [19].

#### 2.2.1. Structural imaging.

Brain morphometry was assessed using the 3D T1-weighted sequences, which were automatically processed with FreeSurfer version 7.2. Cortical thickness measurements were extracted for a total of 34 cortical regions of interest (ROIs) using the Desikan atlas [20]. Additionally, volume measurements were extracted for 7 subcortical ROIs, including the hippocampus, thalamus, amygdala, putamen, globus pallidus, nucleus accumbens, and caudate nucleus. Left and right hemisphere measurements were averaged for all ROIs. Subcortical volumes were adjusted for total intracranial volume (TIV) to account for natural interindividual variability in head sizes using residuals of a least-squares-derived linear regression between each volume and TIV from FreeSurfer [21]. Mean cortical thickness was used as a marker of non-AD-specific neurodegeneration [22]. A cortical signature of AD-specific neurodegeneration was calculated by averaging bilateral entorhinal, inferior temporal, middle temporal, and fusiform thickness, adjusted by cortical surface areas [23]. A cortical signature of brain resilience was calculated by averaging anterior cingulate and temporal pole thickness, adjusted by cortical surface areas [24]. Mean thickness, AD and resilience signature thickness, and hippocampal volume were distributed normally, hence used as continuous measures. Regional cortical thicknesses and volumes were available also at the 5-years follow-up.

#### 2.2.2. Cerebral small vessel disease (SVD).

Markers of cerebral SVD were visually assessed by a neuroradiologist using standard rating scales and according to the Standards for Reporting Vascular Changes on Neuroimaging (STRIVE) [25]. SVD markers included lacunes, identified as 3–15 mm hypointensities in T1 and FLAIR, or hyperintensities in T2; cerebral microbleeds (CMBs) count according to the microbleed anatomical rating scales (MARS) [26]; perivascular spaces count in the centrum semiovale (PVScs) and basal ganglia (PVSbg), according to the Mac Lullich’s rating scale (0–10 vs ≥ 11) [27]. Large infarctions were also visually assessed.

#### 2.2.3. White matter hyperintensity volume.

WMHV was estimated using the Lesion growth algorithm (LGA) as implemented in the Lesion Segmentation Toolbox (LST v3.0.0) in SPM12 (https://www.fil.ion.ucl.ac.uk/spm/), based on probability maps derived from intensity distributions in FLAIR images [28]. WMHV values were normalized for TIV to account for individual differences in head size. The continuous WMHV measure at baseline was categorized intro three groups according to the data distribution: T1 (< 2.7), T2 (2.7–5.5), T3 (> 5.5 mm^3^), with higher values indicating greater small vessel disease burden. Longitudinal information on WMHV was available also at the 5-years follow-up.

#### 2.2.4. White matter microstructural integrity.

Diffusion tensor imaging (DTI) sequences were used to assess white matter microstructural integrity. DTI-derived measure of fractional anisotropy (FA) was extracted using the FMRIB’s Diffusion Toolbox from FSL (v6.0.7.6) (https://fsl.fmrib.ox.ac.uk/fsl/fslwiki) [29]. The FA measure in the white matter used in our analyses is the mean FA of all voxels for each participant computed within the FA skeleton mask, as described in detail elsewhere [30]. The continuous FA measure was categorized into three groups according to the distribution: T1 (< 0.3), T2 (0.3–0.4), T3 (> 0.4), with lower values indicating WM degeneration.

### 2.3. Clinical and fluid biomarkers data

Clinical data were collected by trained research nurses and medical doctors. Detailed assessment procedures have been previously described [17] [31], and are briefly summarized below.

#### 2.3.1. Sociodemographic data.

Participants reported their biological sex at birth and educational attainment, which was categorized into primary and lower secondary schooling (< 9 years of formal education), higher secondary schooling (9 years of schooling or ≤ 2 years of vocational training), and higher education (> 2 years of vocational training or university).

#### 2.3.2. Vascular risk factors and medical conditions.

Smoking was dichotomized into never vs current/former smoking. At-risk alcohol consumption was identified if the person consumed ≥ 100-g alcohol/week (equating to heavy consumption by the National Institute on Alcohol Abuse and Alcoholism) [32]. Physical activity was categorized into inactivity (no activity/sedentary most of the day or irregular lighter walks) vs active (regular non-demanding physical exercise 2–4 times/week, demanding physical activities at least 1 h/week, or regular hard exercise) [32]. Body Mass Index (BMI) was dichotomized into normal vs overweight/obesity if ≥ 25 kg/m^2^. Hypertension was defined as systolic blood pressure ≥ 140 or diastolic blood pressure ≥ 90 mm Hg or current antihypertensive treatment. Cardio- and cerebrovascular conditions (heart diseases such as myocardial infarction, angina pectoris, heart failure, atrial fibrillation, and stroke/TIA) were diagnosed based on examinations, self-report, medication use, or via linkage to the Swedish National Patient Register (NPR), using the International Classification of Diseases-10th edition codes. Prediabetes and diabetes were identified based on self-reported medical history, use of glucose-lowering treatments (diet, oral hypoglycemic agents, or insulin), or fasting/nonfasting blood glucose of ≥ 7.0/11.1 mmol/L [31].

#### 2.3.3. Neuro-psychiatric conditions.

Depression was diagnosed according to the Diagnostic and Statistical Manual of Mental Disorders (DSM), 4th or 5th editions criteria [33]. Dementia was diagnosed according to the DSM 3rd revised criteria merging neuropsychiatric examination and key informant interviews and used only as exclusion criterion in the current study [34]. Medical history of multiple sclerosis, epilepsy, brain cancer, and traumatic brain injury was based on self-reported and close informant interviews on general health.

#### 2.3.4. Cognitive performance.

Cognitive performance was assessed through a detailed test battery encompassing: (1) episodic memory (Memory in Reality–free recall and 12-object delayed recall, and Thurstone’s picture memory), (2) attention and perceptual speed (Figure Identification-PSIF and Digit Span Forward); (3) executive function (Digit Span Backward and Figure Logic); (4) verbal fluency (Controlled Oral Word Association-FAS and semantic fluency from “animals” task); (5) visuospatial abilities (Koh’s Block Test) [31]; (6) a composite score for global cognitive performance generated by averaging the z-scores across the five domains; and (7) MMSE score. Domain-specific tests available at follow-up were standardized into z-scores using the baseline mean and SD [35], and grouped as described previously [31].

#### 2.3.5. Blood biomarkers and apolipoprotein E genotype.

Blood samples were collected from all participants and analysed following the standard lab routines at the Sahlgrenska University Hospital. Indicators of altered lipid metabolism included high triglycerides (≥ 1.7 mmol/L or use of lipid-lowering medication [Anatomical Therapeutic Chemical code C10]), low high-density lipoprotein (HDL) cholesterol (< 1.03 mmol/L in men and < 1.29 mmol/L in women), and low-density lipoprotein (LDL) cholesterol, which was divided into tertiles (T1 ≤ 3; T2 = 3.1–3.9; T3 ≥ 4). Markers of vascular and systemic inflammation were also assessed, including elevated homocysteine (> 13.5 μmol/L) and high C-reactive protein (CRP, ≥ 8 mg/L) [36]. Finally, *APOE* was genotyped, and participants categorized into ε4 allele carriers (one or both ε4 alleles) or non-carriers. For further details on blood and genotyping processing, we refer to the original description of the H70 study cohort [17].

#### 2.3.6. CSF Alzheimer disease biomarkers.

Lumbar puncture was performed in a subset of 286 participants. Biomarkers included and their pathological cut-off values were: β-amyloid 42 (Aβ_42_) ≤ 530 pg/mL, total-tau (t-tau) ≥ 350 pg/mL, and phosphorylated tau at threonine 181 (p-tau) ≥ 80 pg/mL. CSF sampling procedure and biomarkers cut-offs have been previously described in detail [17, 37].

#### 2.3.7. Follow-up status.

Included participant who died during follow-up (Deceased), declined their participation at 5-year re-evaluation (*Dropout*). *Active* participants were those who completed follow-up assessments or were excluded for non-health-related reasons (e.g., migration, technical issues).

### 2.4. Statistical analysis

#### 2.4.1. Identification of the GM patterns through clustering.

We performed cross-sectional clustering using 41 brain ROIs, including 34 cortical regions (measured by cortical thickness) and 7 subcortical structures (measured by volume). We first applied a linear mixed effect regression (LMER), controlling for TIV as fixed effect in subcortical ROIs and subject as a random effect. We then employed unsupervised hierarchical clustering using the random forest algorithm (R software, version 4.0.3, randomForest package, version 4.7–1.1), a method previously used to identify atrophy subtypes in AD, PD and DLB [10, 11, 16, 38]. In our dataset, optimal random forest parameters were: ntree to 6000, mtry to 6, and nodesize to 3. The best cluster solution was established based on a composite score of the Dunn and Calinski-Harabasz indices [39]. For a detailed description of the clustering pipeline see Poulakis et al. [38]. To identify the most discriminating ROIs between the clusters of participants, we calculated Cohen’s d effect sizes based on ROI values averaged across hemispheres within each cluster, comparing Cluster 1 (used as the reference) with Clusters 2 to 5, using independent two-sample t-tests for each ROI with False Discovery Rate (FDR) correction for multiple comparisons (p < 0.05).

#### 2.4.2. Characterization of the GM patterns.

Sociodemographic and genetic variables, vascular risk factors, medical conditions, and blood and neuroimaging biomarkers were compared across clusters (1–5) using Chi-square test for categorical variables, one-way ANOVA for continuous variables normally distributed and Kruskal-Wallis test for continuous variables not normally distributed. To examine the associations between cluster membership (outcome variable, with Cluster 1 as reference) and a range of predictors, we applied multinomial logistic regression (MLR) to estimate Odds Ratios (ORs) and 95% Confidence Intervals (CIs). Predictors included sociodemographic, health-related, lifestyle, cerebrovascular, and fluid biomarkers variables selected based on known dementia risk factors [2] and data availability. We tested four separate MLR models: (1) *Clinical model* included sex (reference = men), educational attainment (reference = primary/lower schooling), any *APOE*-ε4 carriership, MMSE, former/current smoking, at-risk alcohol consumption, physical inactivity, overweigh/obesity, increased triglycerides, increased LDL, reduced HDL, hypertension, heart disease, stroke/TIA, prediabetes and diabetes, depression, and TBI; (2) *Neuroimaging model* included mean FA, WMHV, PVScs, PVSbg, CMBs (absence vs present), lacunes (absence vs present), large infarctions (absence vs present); (3) *Inflammation model* included altered homocysteine and CRP levels; (4) *CSF model* was run only on the subset of participants with available CSF information, and included Aβ42, t-tau, and p-tau biomarkers.

To assess the relationship between cluster allocation and cognitive performance, we conducted six separate Generalized Linear Models (GLMs) using composite scores for global cognition and five cognitive domains as outcomes, and cluster allocation as the predictor. We checked for multicollinearity using the variable inflation factor (VIF), all VIF values were < 1.7, supporting the inclusion of all variables.

A two-sided *p* value < 0.05 indicated statistical significance. All statistical analyses were performed with R software version 4.0.3.

#### 2.4.3. Longitudinal follow-up.

We evaluated five-year longitudinal changes across clusters in seven GM outcomes, cerebrovascular lesions (WMHV), five cognitive domains (attention/perceptual speed, executive functions, verbal fluency, visuospatial ability, global cognition), and MMSE. For GM measures, we included mean cortical thickness, AD-related and resilience cortical signatures thickness, hippocampal volume, and the three regions that most discriminated between clusters, namely the superior frontal, superior parietal, and supramarginal cortices. Longitudinal trajectories were modelled using heterogeneous linear mixed-effects models (via the hlme function from the *lcmm* R package). Each model included fixed effects for timepoint, cluster group, their interaction, years of education, and sex, along with random intercepts and slopes for timepoint nested within individuals. The cluster variable, treated as a five-level categorical factor, was tested via its overall interaction with time. To account for multiple comparisons across outcome models, we applied Bonferroni correction to the minimum p-value of the cluster-by-time interaction in each model.

To explore the association between brain aging clusters of participants and follow-up status at age 75, we performed multinomial logistic regression (*multinom function* from the *nnet* R package). ORs and 95% CIs were computed to assess the likelihood of *Deceased* or *Dropout* vs Active participants (reference) for each cluster compared to Cluster 1.

## Results

### 3.1. Identification of the GM patterns through clustering

**Figure 2:**
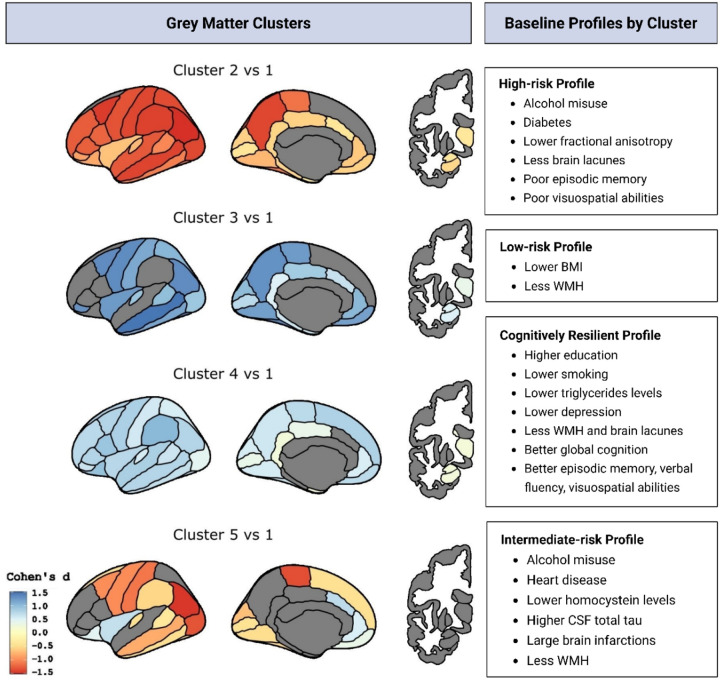
Grey matter clusters and associated brain aging profiles. **Left panel**: cortical maps (left) and subcortical maps (right) display GM differences between each cluster of participants (Clusters 2–5) and Cluster 1 (reference), based on cortical thickness and subcortical volume. Values were averaged across hemispheres. For visualization purposes, we show the left lateral and medial views of cortical regions and the left coronal view of subcortical regions. Cold colours (blue/light blue) indicate greater cortical thickness or subcortical volume, while hot colours (red/yellow) indicate lower ones. The colour bar indicates Cohen’s d effect sizes. **Right panel**: each cluster is characterized by a distinct brain aging profile based on clinical, cognitive, neuroimaging, and biomarkers characteristics. Odds ratios (ORs), β coefficients for cognitive measures, and longitudinal results are reported in the text. Abbreviations: BMI, body mass index; WMH, white matter hyperintensities; CSF, cerebrospinal fluid.

The optimal clustering solution identified five participant clusters, as illustrated in **Supplementary Fig. 1**. Cluster 1 was the largest, comprising 278 participants (37.3%), followed by Cluster 4 (157 participants, 21.1%), Cluster 2 (142 participants, 19.0%), Cluster 3 (121 participants, 16.2%), and Cluster 5 (48 participants, 6.4%). The identified clusters differed significantly in both GM thickness/volume (average values per hemisphere) and regional distribution. Cluster 1 showed no or minimal GM differences from the sample mean and was used as the reference group in subsequent analyses **(Supplementary Fig. 2)**. Figure 2 displays between-cluster brain differences, presenting Cohen’s d effect sizes (Clusters 2–5 vs. Cluster 1) that survived FDR correction (p < 0.05). The three most discriminative ROIs between Clusters 2–5 and Cluster 1, with Cohen’s d values >|1.2| were superior parietal, superior frontal, and supramarginal regions (in that order). This threshold was selected because effect size declined beyond |1.2| (**Supplementary Fig. 3**).

Compared to Cluster 1, Cluster 2 exhibited the most diffuse and severe pattern of reduced cortical thickness and volume (Cohen’s d < −1.2) across numerous ROIs, including frontal (rostral middle frontal, superior frontal, caudal middle frontal, precentral, pars triangularis, pars opercularis), parietal (postcentral, inferior parietal, superior parietal, supramarginal, precuneus), as well as temporal and occipital regions. Cluster 3 had thicker cortex (Cohen’s d > 1.2) in more posterior regions, particularly across temporo-parietal (middle temporal, inferior temporal, inferior parietal, paracentral, precuneus, insula) and occipital (cuneus, lingual, fusiform, lateral occipital) areas. Cluster 4 had a more diffuse but less pronounced pattern of thicker cortex (Cohen’s d > 1.04). Finally, Cluster 5 showed a mixed pattern, with thinner cortex in frontoparietal (superior frontal, caudal middle frontal, precentral, postcentral, supramarginal, inferior parietal, paracentral) and occipital (lateral occipital) regions (Cohen’s d < −1.2), alongside thicker cingulate, insular, and orbitofrontal cortex (Cohen’s d > 0.4). **Supplementary Fig. 4** reports cortical thickness (mm) and subcortical volume values (mm^3^), averaged across hemispheres, for all ROIs for the five participants’ clusters.

### 3.2. Baseline participant characteristics

No significant differences across the identified clusters were observed in baseline clinical characteristics and *APOE* status. Regarding neuroimaging features, there were significantly differences among clusters in mean cortical thickness, FA, and WMHV (all *p* < 0.001), as well as in the prevalence of large infarctions (p = 0.043), as reported in Table 1. There were no differences in CSF AD biomarkers (**Supplementary Table 1**). Baseline characteristics did not differ between participants with CSF data (n = 286) vs. those without (n = 460) (**Supplementary Table 2**).

### 3.3. Characterization of GM patterns through baseline and longitudinal regression analyses

**Figure 3:**
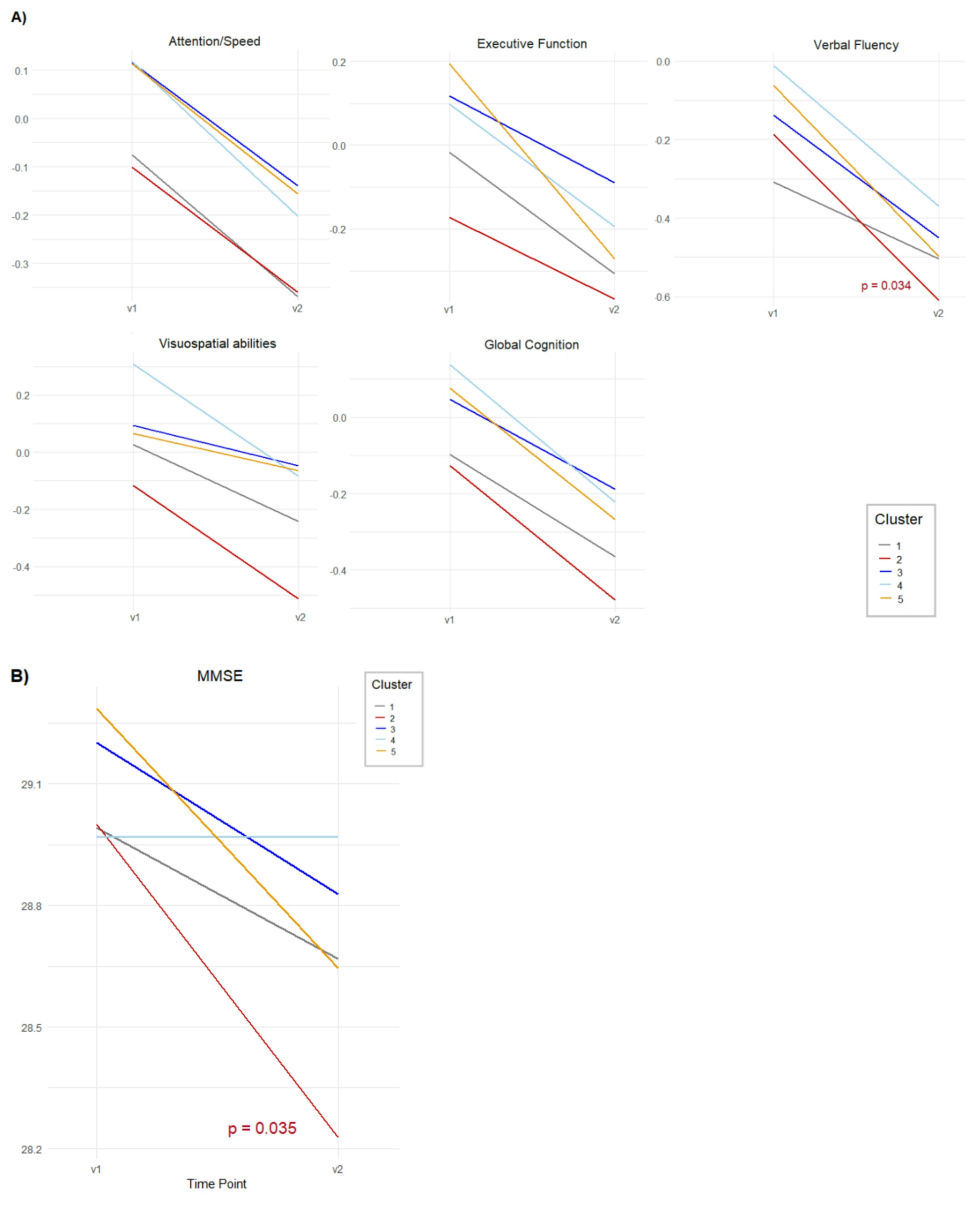
Longitudinal changes in cognitive performance across clusters over five years. Trajectories represent changes from baseline (v1) to follow-up (v2) for each cluster in **(A)** five cognitive domains: Attention/Speed, Executive Function, Verbal Fluency, Visuospatial Abilities, Global Cognition, and **(B)** MMSE.

**Figure 4:**
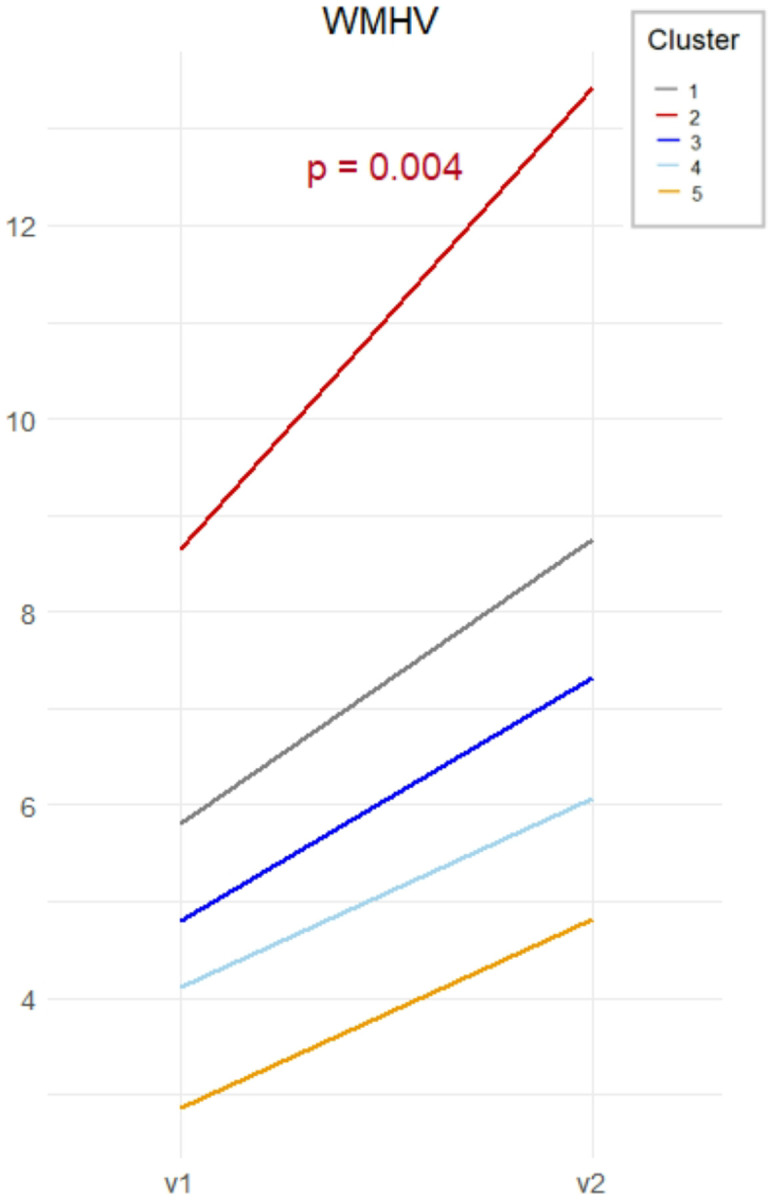
Longitudinal changes in white matter hyperintensities volume (WMHV) across cluster over five years. Trajectories represent changes in WMHV from baseline (v1) to follow-up (v2) for each cluster.

Using Cluster 1 as the reference, we estimated the odds of participants belonging to Clusters 2–5 by applying the four previously described models (Clinical, Neuroimaging, Inflammation, CSF). Figure 2 shows the defining features of each brain aging profile, and full model estimates (odds ratios and 95% confidence intervals) are reported in **Supplementary Table 3**. Generalized linear models revealed between-cluster differences in baseline cognitive performance (**Supplementary Table 4**), while mixed-effects models were used to explore trajectories in cognition and brain measures (Figs. 3 and 4; **Supplementary Table 5**). Based on the identified GM patterns, their clinical and cognitive correlates, and longitudinal changes, we defined four brain aging profiles.

#### 3.3.1. Cluster 1: Reference group.

Cluster 1 was used as the reference category across all regression analyses. It comprised the largest proportion of participants (37.3%) and showed no or minimal GM differences from the sample mean.

#### 3.3.2. Cluster 2: High-risk profile.

As summarized in Fig. 2, compared to Cluster 1, participants in Cluster 2 had significantly higher odds of risk alcohol consumption (OR = 1.83, 95% CI [1.13, 2.97]), diabetes (OR = 2.54, 95% CI [1.27, 5.06]), lower fractional anisotropy values (OR = 0.46, 95% CI [0.26, 0.83]), and lower lacunes (OR = 0.35, 95% CI [0.14, 0.84]). They also showed significantly poorer performance in episodic memory (β = − 0.190, p = 0.014) and visuospatial abilities (β = − 0.209, p = 0.044) at baseline.

Longitudinally, Cluster 2 had a significant decline in verbal fluency (β_slope_ = − 0.226, p = 0.008) and MMSE scores (β_slope_ = − 0.448, p = 0.035) (Fig. 3A and 3B), along with a steeper increase in WMHV (β_slope_ = 1.843, p = 0.004) (Fig. 4). There were no significant longitudinal changes in GM measures. Cluster 2 was borderline associated with greater mortality during follow-up, although it did not reach statistical significance (OR = 2.18, 95% CI [0.93, 5.11]) (**Supplementary Table 6)**.

#### 3.3.3. Cluster 3: Low-risk profile.

Participants in this group were significantly less likely to be overweight or obese (OR = 0.57, 95% CI [0.35, 0.94]). They also showed lower odds of having moderate (OR = 0.45, 95% CI [0.26, 0.77]) or high (OR = 0.50, 95% CI [0.27, 0.91]) WMHV (Fig. 2). No significant longitudinal changes in this group were observed.

#### 3.3.4. Cluster 4: Cognitively resilient profile.

Participants in Cluster 4 were more likely to have a higher educational level (OR = 2.52, 95% CI [1.08, 5.87]), and less likely to be current or former smokers (OR = 0.62, 95% CI [0.40, 0.95]). They also had lower odds of elevated triglyceride levels (OR = 0.55, 95% CI [0.32, 0.95]) and were less likely to have a history of depression (OR = 0.17, 95% CI [0.05, 0.56]). Individuals in this cluster were also less likely to have greater WMHV (OR for tertile 2 = 0.54, 95% CI [0.33, 0.88]; OR for tertile 3 = 0.65 95% CI [0.38, 1.12]) and had a lower likelihood of exhibiting brain lacunes (OR = 0.18, 95% CI [0.05, 0.63]). This group performed significantly better than Cluster 1 across multiple cognition domains at baseline, including global cognition (β = 0.158, p = 0.010), episodic memory (β = 0.155, p = 0.040), verbal fluency (β = 0.237, p = 0.006), and visuospatial abilities (β = 0.222, p = 0.026) (Fig. 2). No significant longitudinal changes were observed in this cluster.

#### 3.3.5. Cluster 5: Intermediate-risk profile.

Participants in Cluster 5 were significantly more likely to have risk alcohol consumption (OR = 2.09, 95% CI [1.05, 4.16]) and heart disease (OR = 3.44, 95% CI [1.48, 8.01]) (Fig. 2). They also showed a higher likelihood of presenting large infarctions (OR = 17.44, 95% CI [1.95, 156.15]), but a reduced odds of falling into the highest tertile of WMHV (OR = 0.23, 95% CI [0.08, 0.64]). In addition, they had lower homocysteine levels (OR = 0.40, 95% CI [0.19, 0.84]), and increased odds of elevated total tau levels (OR = 3.35, 95% CI [1.22, 9.14]). No significant longitudinal changes in this group were observed. Similar to Cluster 2, Cluster 5 was borderline associated with greater mortality during follow-up, although it did not reach statistical significance (OR = 1.67, 95% CI [0.44, 6.28]) (**Supplementary Table 6)**.

## Discussion

In this longitudinal, population-based study, we assessed a cohort of cognitively healthy 70-year-old individuals using a data-driven MRI approach to identify specific cortical and subcortical grey matter (GM) patterns, their clinical correlates, and their evolution over a 5-year follow-up. We identified five clusters of participants based on distinct GM profiles, primarily differentiated by variations in the superior frontal, superior parietal, and supramarginal gyri. Compared to Cluster 1 (reference), Cluster 2 exhibited diffusely lower frontoparietal cortical GM and was associated with higher odds of diabetes, risk alcohol consumption, disrupted white matter integrity, cerebrovascular lesions and poorer episodic memory and visuospatial performance. Clusters 3 and 4 showed higher GM values in several brain regions and were associated with a healthier cardiometabolic profile, lower depression rates, higher educational attainment, and reduced brain small vessel disease. Cluster 4 also had superior cognitive performance across multiple domains. Cluster 5, the smallest group, had a mixed GM and cerebrovascular pattern and was characterized by higher odds of risk alcohol consumption, heart disease, and elevated CSF tau, but lower inflammation levels. Longitudinally, GM measures did not differ significantly across clusters, but Cluster 2 showed greater WMHV increase and steeper decline in MMSE and verbal fluency over time. No differences were observed in *APOE* status or CSF AD biomarkers across clusters.

Previous MRI studies have shown that normal brain aging is associated with widespread reductions in cortical thickness and volumes, particularly in the prefrontal and parietal regions [3, 40, 41]. Advanced brain aging, defined as a significant deviation from typical age-related trajectories, has been linked to more pronounced GM loss in these same areas [6]. Preferential involvement of the superior frontal [40] and supramarginal gyri [41] has also been reported, which emerged as key discriminative regions in our study. These frontoparietal regions play a critical role in higher-order cognitive functions [42], and their degeneration may impair cognition [3]. This relationship was evident in Cluster 2, which showed less frontoparietal GM and lower cognitive performance, whereas Cluster 4 was characterized by preserved frontoparietal structures and better cognition. Our longitudinal data revealed no significant differences in GM changes between clusters over time, consistent with previous studies showing similar trajectories among cognitively healthy individuals, even across groups with different risk profiles [13].

Few structural imaging studies have applied GM clustering approaches in large cohorts of cognitively healthy adults. Recently, Skampardoni et al. [13] used advanced deep learning methods to identify a resilient brain aging group characterized by preserved brain volumes, lowest cardiovascular risk factors, and highest baseline cognition, closely resembling Clusters 3 and 4 in our study. They also described a vascular aging profile, which mirrors our Cluster 2, marked by lower peri-Sylvian, orbitofrontal, and prefrontal thickness, higher cardiovascular risk factors, increasing WMH burden, and poorer cognition. Similarly, in our previous work [16] frontoparietal GM profiles and trajectories were also linked to cerebrovascular burden, as measured with White Matter Signal Abnormalities (WMSA, consisting of hypointensities), and cognitive decline [16]. Capogna and colleagues [15] applied clustering methods based on changes in cortical thickness, surface area, and subcortical volume. Subtypes based on cortical thickness and area were associated with bilateral decline in temporal and inferior parietal regions, rather than frontal areas. Cognitive outcomes were linked to cortical area changes, while AD biomarkers were associated with subcortical volume. Discrepancies with our findings may reflect cohort differences, inter-individual variability, and variation in cognitive and imaging metrics across merged datasets.

The clinical characterization of participants across GM-based clusters highlighted the relevance of levels vascular and psychosocial factors in shaping brain health [43, 44]. Cluster 2, marked by lower GM, was associated with diabetes and risk alcohol consumption, and Cluster 5 with increased cardiovascular risk. Diabetes has been linked to reduced cortical thickness across multiple lobes, independent of socioeconomic factors and comorbidities, with higher HbA1c driving this effect [45]. It has also been associated with dementia and cognitive decline, possibly through neurodegenerative mechanisms unrelated to small vessel disease or AD biomarkers [46], and with microstructural white matter alterations [47], consistent with the reduced fractional anisotropy observed in Cluster 2. Similarly, the favourable cerebrovascular and inflammatory profile (lower WMH and homocysteine, in line with previous findings linking systemic inflammation and cerebrovascular lesions [36, 48]) and anterior cingulate preservation in Cluster 5 might have counteracted the effects of reduced GM and risk factors, perhaps safeguarding cognition through brain maintenance (i.e., the preservation of cognitive function and neural resources) and/or cognitive reserve (i.e., the brain’s ability to maintain cognitive function despite aging or pathology) [49, 50]. Similarly, alcohol risk consumption and higher cardiovascular risk have both been associated with reduced cortical thickness and poorer cognitive performance [51, 52]. In contrast, Clusters 3 and 4 showed preserved GM patterns, favourable metabolic and lifestyle profiles, and fewer radiological signs of brain small vessel disease, a known contributor to cortical atrophy and cognitive decline [53]. Cluster 3 was associated with lower body mass index, aligning with evidence indicating that BMI stability supports brain health [54]. Cluster 4 showed lower prevalence of smoking and higher educational attainment. Smoking has been associated with dose-dependent cortical thinning [55], while higher education with increased mean cortical thickness across the cortex, independent of vascular risk factors [56]. Finally, in the analysis of AD CSF biomarkers conducted in a subsample of our population, we found a high prevalence of Aβ positivity, but no significant differences across subgroups with distinct GM patterns. These findings possibly indicate that amyloid pathology may not be the primary driver of structural brain alterations in healthy older adults, in line with previous studies suggesting that tau pathology is more strongly associated with both cortical thinning and cognitive decline [57].

Our study presents several strengths. First, the use of an unsupervised, data-driven clustering approach in a general population sample allowed the identification of previously unrecognized GM patterns, revealing substantial brain heterogeneity in cognitively healthy older adults. Second, the strict age-homogeneity of the sample minimized confounding effects of age. Third, the study leverages a large, longitudinal, well-characterized population-based cohort with extensive clinical, lifestyle, and imaging data. Limitations include reduced generalizability due to the age-specific sample and the possibility of residual confounding. A ceiling effect may have limited the inclusion of individuals with more severe findings, who were likely classified as cognitively impaired and thus excluded. Follow-up data were available only for a subset, possibly representing a healthier segment of the cohort, which may explain why some cognitive differences detected in the cross-sectional GLM do not appear as differences in intercepts in the longitudinal mixed model. Lastly, the limited availability of AD and non-AD neurodegeneration biomarkers restricted assessment of their association with GM patterns.

In conclusion, using a data-driven MRI clustering approach in a population-based cohort, we identified five subgroups with distinct cortical and subcortical GM patterns, each associated with specific clinical and cognitive profiles. Vascular and psychosocial factors, such as diabetes, cardiovascular diseases, alcohol consumption, obesity, smoking, and education, emerged as key determinants of GM integrity, influencing both cognitive performance and its longitudinal trajectory. Further research is needed to clarify the underlying biological mechanisms and to determine whether targeting these modifiable risk factors can effectively preserve brain structure and function through late life.

## Supplementary Files

This is a list of supplementary files associated with this preprint. Click to download.

- LorenzonSupplementaryMaterialsubmission.docx

## Figures and Tables

**Table 1:** Baseline characteristics of participants from the Gothenburg H70– Birth Cohort 1944 by cluster.

	Cluster 1 (N = 278)	Cluster 2 (N = 142)	Cluster 3 (N = 121)	Cluster 4 (N = 157)	Cluster 5 (N = 48)	Total (N = 746)	*p* *
Sociodemographic and genetic factors							
Age, years	70.8 ± 0.3	70.9 ± 0.4	70.8 ± 0.4	70.8 ± 0.3	70.8 ± 0.3	70.8 ± 0.3	0.561
Sex							0.423
Men	131 (47.1)	79 (55.6)	54 (44.6)	68 (43.3)	21 (43.8)	353 (47.3)	
Women	147 (52.9)	63 (44.4)	67 (55.4)	89 (56.7)	27 (56.2)	393 (52.7)	
Education							0.423
Primary/lower secondary	36 (12.9)	19 (13.4)	9 (7.4)	11 (7.1)	7 (14.6)	82 (11.0)	
Higher secondary	137 (49.3)	75 (52.8)	62 (51.2)	74 (47.4)	20 (41.7)	368 (49.4)	
Higher education	105 (37.8)	48 (33.8)	50 (41.3)	71 (45.5)	21 (43.8)	295 (39.6)	
*APOE* status (any ɛ4 carriers)	85 (31.2)	52 (36.9)	34 (29.1)	52 (34.2)	17 (35.4)	240 (32.9)	0.855
MMSE score	29.1 ± 1.2	28.9 ± 1.3	29.2 ± 1.0	29.2 ± 1.1	29.1 ± 1.4	29.1 ± 1.2	0.561
Cardiovascular risk factors							
Current/former smoker	179 (64.4)	96 (68.1)	71 (59.2)	81 (51.9)	29 (60.4)	456 (61.4)	0.153
Alcohol risk consumption	80 (28.8)	58 (40.8)	36 (30.0)	41 (26.3)	20 (41.7)	235 (31.6)	0.120
Physical inactivity	12 (4.5)	5 (3.6)	2 (1.7)	3 (1.9)	3 (6.4)	25 (3.4)	0.509
Overweight (BMI ≥ 25 kg/m^2^)	152 (58.9)	85 (64.4)	54 (44.6)	84 (56.4)	25 (54.3)	400 (56.7)	0.120
Elevated triglycerides	108 (39.0)	66 (46.5)	42 (35.0)	42 (26.9)	15 (31.2)	273 (36.7)	0.077
Reduced HDL cholesterol	34 (12.3)	16 (11.3)	11 (9.2)	19 (12.3)	6 (12.5)	86 (11.6)	0.919
LDL cholesterol							0.498
T1 (< 3)	92 (33.2)	56 (39.4)	46 (38.3)	46 (29.7)	15 (31.2)	255 (34.4)	
T2 (3.1–3.9)	98 (35.4)	49 (34.5)	34 (28.3)	61 (39.4)	13 (27.1)	255 (34.4)	
T3 (≥ 4)	87 (31.4)	37 (26.1)	40 (33.3)	48 (31.0)	20 (41.7)	232 (31.3)	
Hypertension (≥ 140/90mmHg)	196 (70.5)	103 (72.5)	83 (68.6)	105 (67.3)	32 (66.7)	519 (69.7)	0.919
Medical conditions							
Heart disease	45 (16.2)	21 (14.8)	21 (17.4)	31 (19.9)	14 (29.2)	132 (17.7)	0.393
Stroke/TIA	22 (7.9)	13 (9.2)	7 (5.8)	11 (7.0)	4 (8.3)	57 (7.6)	0.919
Diabetes							0.077
Normoglycemia	114 (41.0)	50 (35.2)	55 (45.5)	75 (48.1)	19 (39.6)	313 (42.0)	
Prediabetes	127 (45.7)	55 (38.7)	55 (45.5)	64 (41.0)	22 (45.8)	323 (43.4)	
Diabetes	37 (13.3)	37 (26.1)	11 (9.1)	17 (10.9)	7 (14.6)	109 (14.6)	
Depression (major/minor)	28 (10.1)	12 (8.5)	10 (8.3)	4 (2.6)	5 (10.4)	59 (7.9)	0.244
Traumatic brain injuries	85 (30.6)	50 (35.2)	38 (31.4)	55 (35.0)	14 (29.2)	242 (32.4)	0.919
Blood biomarkers							
Homocysteine > 13 μmol/L	110 (40.3)	57 (40.4)	40 (33.9)	54 (35.8)	10 (21.3)	271 (37.1)	0.283
C-reactive protein ≥ 8 mg/L	18 (6.6)	15 (10.7)	10 (8.5)	7 (4.6)	6 (12.5)	56 (7.7)	0.393
Neuroimaging biomarkers							
Mean cortical thickness, mm	2.3 ± 0.0	2.2 ± 0.1	2.5 ± 0.0	2.4 ± 0.0	2.3 ± 0.0	2.4 ± 0.1	< 0.001*
Average Fractional Anisotropy							< 0.001*
T1 (< 0.3)	90 (34.0)	71 (53.0)	26 (22.8)	34 (22.5)	16 (34.8)	237 (33.4)	
T2 (0.3–0.4)	85 (32.1)	37 (27.6)	35 (30.7)	61 (40.4)	19 (41.3)	237 (33.4)	
T3 (> 0.4)	90 (34.0)	26 (19.4)	53 (46.5)	56 (37.1)	11 (23.9)	236 (33.2)	
WMHV, mm^3^							< 0.001*
T1 (< 2.7)	78 (28.5)	33 (24.1)	57 (47.5)	59 (38.6)	17 (35.4)	244 (33.3)	
T2 (2.7–5.5)	103 (37.6)	35 (25.5)	35 (29.2)	51 (33.3)	21 (43.8)	245 (33.5)	
T3 (> 5.5)	93 (33.9)	69 (50.4)	28 (23.3)	43 (28.1)	10 (20.8)	243 (33.2)	
Cerebral microbleeds	35 (12.8)	19 (13.5)	13 (10.7)	13 (8.3)	1 (2.1)	81 (10.9)	0.279
Lacunes (3–15 mm)	28 (10.1)	11 (7.8)	6 (5.0)	4 (2.5)	3 (6.2)	52 (7.0)	0.118
Large infarctions	3 (1.1)	7 (5.0)	1 (0.8)	1 (0.6)	3 (6.2)	15 (2.0)	0.043*
Enlarged PVS CS							0.919
0–10	75 (27.4)	34 (24.1)	33 (27.3)	43 (27.4)	11 (22.9)	196 (26.5)	
≥11	199 (72.6)	107 (75.9)	88 (72.7)	114 (72.6)	37 (77.1)	545 (73.5)	
Enlarged PVS BG							0.192
0–10	220 (80.3)	99 (70.2)	101 (83.5)	125 (79.6)	38 (79.2)	583 (78.7)	
≥11	54 (19.7)	42 (29.8)	20 (16.5)	32 (20.4)	10 (20.8)	158 (21.3)	

*Note*. Data are presented as Mean ± Standard deviations for continuous variables or number (percentage) for categorical variables.

Abbreviations: *APOE*-ɛ4, apolipoprotein E gene-ɛ4 allele; MMSE, Mini-Mental State Examination; BMI, body mass index; HDL, High-density lipoprotein; LDL, Low-density lipoprotein; T, tertile; TIA, transient ischemic attack; WMHV, white matter hyperintensity volume; PVS, perivascular spaces; CS, centrum semiovale; BG, basal ganglia.

Missing data: Education (n = 1), *APOE*-ɛ4 (n = 16), MMSE (n = 4), Smoking (n = 3), Alcohol consumption (n = 2), Physical activity (n = 15), BMI (n = 2), Elevated triglycerides (n = 3), Reduced HDL cholesterol (n = 4), LDL cholesterol (n = 4), Hypertension (n = 1), Heart disease (n = 1), Prediabetes/diabetes (n = 1), Depression (n = 2), Homocysteine (n = 16), C-reactive protein (n = 16).

WMHV (n = 3), Cerebral microbleeds (n = 6), Lacunes (n = 3), Large infarctions (n = 4), Enlarged PVS Centrum semiovale (n = 5), Enlarged PVS Basal ganglia (n = 5).

* p value < 0.05.

## Data Availability

Data are from the H70 Gothenburg Study project (https://www.gu.se/en/research/the-gothenburg-h70-birth-cohort-study). Access to this original data is available to the research community upon approval by the H70 Gothenburg Study coordination group. Applications for accessing these data can be submitted through Eric Westman (eric.westman@ki.se) at the Department of Neurobiology, Care Sciences and Society, Karolinska Institutet. Code for data analyses is available on request from the corresponding authors, Giulia Lorenzon (giulia.lorenzon@ki.se), and Eric Westman (eric.westman@ki.se).

## Abbreviations

GM: Grey Matter
WM: White Matter
ROI(s): Region(s) of Interest
TIV: Total Intracranial Volume
AD: Alzheimer’s Disease
PD: Parkinson’s Disease
DLB: Dementia with Lewy Bodies
CSF: Cerebrospinal Fluid
OR: Odds Ratio
CI: Confidence Interval
β: Beta coefficient (regression coefficient)
p: p–value (statistical significance)
MRI: Magnetic Resonance Imaging
3D: Three–Dimensional
TFE: Turbo Field Echo
FLAIR: Fluid–Attenuated Inversion Recovery
DTI: Diffusion Tensor Imaging
BOLD: Blood–Oxygen–Level–Dependent
AD: **specific cortical signature**–Average thickness in specific AD–related cortical regions
WMHV: White Matter Hyperintensity Volume
FA: Fractional Anisotropy
PVScs: Perivascular Spaces in Centrum Semiovale
PVSbg: Perivascular Spaces in Basal Ganglia
CMB(s): Cerebral Microbleed(s)
SVD: Small Vessel Disease
STRIVE: Standards for Reporting Vascular Changes on Neuroimaging
MMSE: Mini–Mental State Examination
PSIF: Perceptual Speed Identification Figure (from Figure Identification test)
DSM: Diagnostic and Statistical Manual of Mental Disorders
TIA: Transient Ischemic Attack
BMI: Body Mass Index
APOE: Apolipoprotein E
Aβ42: Beta–Amyloid 1–42
t: **tau**–Total Tau protein
p: **tau**–Phosphorylated Tau protein (at threonine 181)
HDL: High–Density Lipoprotein
LDL: Low–Density Lipoprotein
CRP: C–Reactive Protein
LMER: Linear Mixed–Effects Regression
MLR: Multinomial Logistic Regression
GLM: Generalized Linear Model
VIF: Variance Inflation Factor
FDR: False Discovery Rate
H70: Gothenburg H70 Birth Cohort Study
NPR: National Patient Register
ICD: International Classification of Diseases
HbA1c: Hemoglobin A1c (glycated hemoglobin)
